# Aging With Cerebral Palsy: A Photovoice Study Into Citizenship

**DOI:** 10.3389/fneur.2021.729509

**Published:** 2021-08-31

**Authors:** Vera G. van Heijningen, Mieke Cardol, Heidi J. M. van Heijningen-Tousain, Daniëlla M. Oosterveer, Frederike van Markus-Doornbosch, Jane N. T. Sattoe, Menno van der Holst, Sander R. Hilberink

**Affiliations:** ^1^Research Centre Innovations in Care, Rotterdam University of Applied Science, Rotterdam, Netherlands; ^2^Basalt Rehabilitation, Hague/Leiden, Netherlands; ^3^Department of Orthopaedics, Rehabilitation and Physical Therapy, Leiden University Medical Center, Leiden, Netherlands

**Keywords:** cerebral palsy, citizenship, aging, adults, photovoice, qualitative research

## Abstract

**Background:** Adults with cerebral palsy (CP) may experience an increasing impact of their disability on daily life and this may interfere with their citizenship. Citizenship is a layered construct. Next to formal and theoretical significations, and civil rights acts such as the UN Convention on the Rights for Persons with Disabilities (CRPD), the meaning of citizenship is formed by the person themselves. The present study aimed to gain insight into what citizenship means for adults with CP 40 years or older and what is needed to support and pursue their citizenship to improve person-centered rehabilitation which can facilitate this process.

**Methods:** Adults with CP (>40 years) without intellectual disability were recruited from medical records of a large rehabilitation center to participate in a qualitative study using the photovoice method. Participants were asked to take photos of objects or life situations that constituted citizenship for them; these photos were then the prompts for the semi-structured interviews that were held face-to-face at their homes. Background and clinical characteristics were gathered using a short face-to-face questionnaire. Data were analyzed through inductive thematic analysis.

**Results:** Nineteen adults participated [mean age (SD) 57.8 (9.4) years (range 44–79), six men]. From the analysis four themes emerged: (a) Meanings of citizenship; (b) Citizenship: Facilitator and barriers; (c) Paradoxes of support and participation; and (d) Future. Furthermore, next to the ability to participate in society without restrictions, sense of belonging was reported to be an important aspect of “meanings of citizenship.” The physiotherapist was perceived as an important health professional to maintain physical activity and deal with the impact of aging with CP on daily activities. Complex healthcare and support services regulations and aging affected citizenship negatively.

**Conclusion:** Middle-aged and older adults with CP view citizenship as the ability to participate and belong in society. To optimize their citizenship the challenges and individual needs must be seen and supported by person-centered rehabilitation and support services. Simplification of complex healthcare and services regulations can further improve citizenship.

## Introduction

Cerebral palsy (CP) is the largest population in pediatric rehabilitation (1) and has an estimated incidence of 2.0–3.5 per 1,000 live births (2, 3). Seventy-five percent of persons with CP are adults, of which approximately 60% is older than 40 years (4). In the past two decades increasing attention has been paid to the adolescents and (young) adult population with CP, research in the middle-aged and older adult population with CP is scarce (5).

Fatigue and increasing pain are the most common problems reported by adults with CP (2, 6–10). As a result, the perceived impact of CP on daily activities increases with age with mobility and self-care declining (7), and community participation becoming more difficult (11, 12). This increasing impact of aging with CP complicates the delicate balance between demands of daily life and available energy levels and makes demands on the adaptability of adults with CP. This process also affects one's citizenship.

In 2006, the United Nations adopted the Convention on the Rights of Persons with Disabilities (CRPD) (13). The CRPD “is intended as a human rights instrument with an explicit, social development dimension. It adopts a broad categorization of persons with disabilities and reaffirms that all persons with all types of disabilities must enjoy all human rights and fundamental freedoms” (14). As such, it is a civil rights act, advocating equal citizenship for persons with disabilities in terms of autonomy and respect for human diversity. Citizenship is a layered construct: it refers to (the ability to exercise) civil rights, (15) making a contribution to society (often narrowed to paid employment), (16) and to democratic practice and identity (17). These aspects concur with the review by Waldschmidt and Sépulchre, (18) who described three ambivalent citizenship roles for persons with disabilities: social citizenship, autonomous citizenship and political citizenship. The social citizenship role stresses the value of making a contribution to society; those citizens who cannot meet this value can rely on the solidarity of the welfare state. This solidarity, however, can result in stigmatization (e.g., high social control and minimum income) and increases the risk of marginalization. The autonomous citizenship role conflicts with the practice that all citizens are interdependent (i.e., dependent on one another), but in the case of disability, this interdependence is more pronounced and labeled as dependence. This mechanism hampers the autonomous citizenship role. Lastly, the political citizenship role refers to representation and involvement in decision-making. Still, those citizens who utilize healthcare and support services often have little influence in how this support is legislated, implemented and operationalized; hence their political citizenship role is restrained (18). The way in which adults with disabilities experience their citizenship – and how policy environments impact this – may vary internationally due to differences in support and healthcare systems. Dutch adults with disabilities define citizenship as equality and diversity (19). Australian young adults with CP identify four aspects of citizenship: contribution to society, inclusion, equal opportunities, and a context without barriers (20). Yet the lived experiences of older adults with CP regarding their citizenship are unknown.

Citizenship is closely related to rehabilitation outcomes such as community participation and fulfilling social roles (21, 22). However, rehabilitation and support services are not always sensitive to the context, expertise and autonomy of adults with CP (12). Person-centered rehabilitation addresses patients' needs, preferences, experiences or knowledge and cultural values, as well as their history and biography (23). To optimize person-centered rehabilitation and support services for adults with CP, the present study aims to gain insight into what constitutes citizenship for middle-aged and older adults with CP and how they experience their citizenship.

## Methods

### Design

This study used a qualitative design, based on the photovoice method applying photos and semi-structured interviews. Wang and Burris described photovoice as “a process by which people can identify, represent, and enhance their community through a specific photographic technique” [(24), p.369]. Photovoice offers an opportunity to share experiences and viewpoints with others whose decisions affect their lives, (25) captures the non-verbal experiences of participants (26) and allows participants to set the interview agenda to ensure that the interview connects with their lived experiences. Box 1 shows how the photovoice method was applied in the present study. The local Medical Research Ethics Committee Leiden The Hague Delft in the Netherlands granted ethical approval (NL72958.058.20). All participants signed informed consent themselves.

Box 1 **The application of the Photovoice method in the present study.**

To understand how middle-aged and older persons with CP experience their citizenship, the photovoice method was used. Photovoice studies are often conducted with persons who belong to traditionally marginalized groups, who are seldom heard. With photovoice, persons become involved and have the chance to share their knowledge and views (25). The first author conducted the interviews, prior to data collection she tested the photovoice method and interview with the last author to become familiarized with this method. In this study, the photographs formed the prompt for the interview and were not included in the thematic analyses. After receiving the signed informed consent to participate in the study, participants were contacted by telephone to explain and clarify the method, to answer questions and to schedule an interview. Participants had two to four weeks to take these photos. The researcher also signed the informed consent form and sent a copy to the participant. Participants were asked to take 4–10 photographs of: (1) places, people, situations or objects, activities that were important in their life and that showed how they perceived the world; and (2) examples/situations of how they shaped their citizenship, e.g. in what ways they participated in society. When it was not possible for a participant to take photographs themselves, it was allowed to use photos or images from magazines or from the Internet, or ask another person to take photos. It was stressed that the images should depict what the participant would have photographed. Participants were asked to mail the photos to the researcher by email. When received, the photographs were printed and labeled so the participant could easily choose the photos they wanted to talk about in the interview. To avoid any influence of the labeling on the participant's choice for the ranking of the photos during the interview, each photo was assigned to a color. Not all photos were discussed during the interviews, participants were free to say if they had used enough photos to tell their story. The face-to-face interview took place at their home (one interview was held online due to Covid-19 safety measures). At the start of the interview, all photo were spread out on the table and the participant was invited to choose a photo with which he/she wanted to start. Participants were asked: (1) “What is your story with this photo?”; and (2) “Can you tell me more about how this photo relates to citizenship?.” During the interviews the participants were encouraged to tell more about it by asking open-ended questions about the photo and following the story, to reveal a more in-depth look at what the photo meant to them.

### Participants

Adults with CP were recruited at a large rehabilitation center. Eligibility was checked via the electronic medical records. The following inclusion criteria were applied: (1) a documented diagnosis of CP; (2) aged > 40 years; and (3) good comprehension of the Dutch language. The ability to communicate verbally was not an inclusion criterium. Adults were excluded in case of: (1) a documented severe learning disability (IQ<70); and (2) severe comorbidity (including depression, severe somatic disorders). On behalf of the research group, an invitation was sent by the participating rehabilitation center to eligible participants. This invitation included an explanation of the study and an informed consent form. The first author was available by phone to answer any questions regarding the study. After the participant signed the informed consent, the first author made an appointment for the interview.

### Data Collection

Prior to the interviews, participants were asked during a telephone call to take photos of places, people, situations, objects, or activities expressing their meanings, opinions and/or experiences of citizenship. Participants could also use existing photos, images from the internet, or ask others to take photos for them. These photos were used only as prompts for the interview and were not included in the analyses. In the face-to-face interview lasting 60–90 minutes, participants showed their photos and related them to their meanings of and experiences of citizenship. Participants were asked what they would need to optimize their citizenship in the present and in the future. Citizenship was introduced to the participants as a multi-layered construct (Appendix I). The interviews were conducted by the first author and were audio-recorded, transcribed verbatim and anonymized for analysis. The interviews were conducted from September 15 to December 9, 2020.

To describe the sample, a short face-to-face questionnaire assessed background characteristics (sex, age, educational level, work / daily activities, living situation, marital status, having children, support at home). Educational level was classified as low (prevocational practical education or lower), medium (prevocational theoretical education and upper secondary vocational education), or high (secondary education, higher education, and university) (27). Work/daily activities were assessed by the question: Do you have a job? [(1) yes, paid; (2) yes, unpaid; (3) no, daycare facility; and (4) no, no job or daycare facility]. Participants were asked if they received support or care at home (no/yes). The first author assessed the clinical characteristics of CP, including gross motor function (Gross Motor Function Classification System; GMFCS), (28) manual ability (Manual Ability Classification System; MACS), (29) and CP type (spastic, non-spastic) and laterality (unilateral, bilateral). Both GMFCS and MACS are a five-level classification system (I = least severely affected to V = most severely affected).

### Data Analysis

Thematic analysis was applied. Thematic analysis is a practical, step-by-step tool for recognizing patterns in qualitative data (30). Transcriptions were read and re-read to become familiar with the data. The data were then divided into fragments and inductively analyzed and coded. The codes were organized into groups and a potential theme was formed for each group. It was then verified that the themes and groupings worked for the coded fragments and for the complete data set as well. Saturation was reached when an interview did not reveal new codes. As a final step, the main themes and sub-themes were defined, named and linked. To illustrate the themes, quotes made by the participants that corresponded to the specific theme were used. The first four interviews were analyzed by two authors (VvH, JS), codes were compared, and differences were discussed and converged with the second author (MC). The other interviews were analyzed by one author (VvH), under supervision of JS and SH. Final codes and relations between codes were discussed between VvH, SH and MC. Data were analyzed using Atlas.ti version 9.0 for Windows.

### Development of Implementation Materials

Six adults with CP, who did not participate in the study, were asked in three meetings, to reflect on the study results to help future adults with CP and (rehabilitation) professionals with these themes. They proposed how to translate the findings into easily accessible formats. They recommended developing an infographic and making a short YouTube video. Although the development of these implementation materials goes beyond the scope of this study and therefore is not described in this publication, the developed infographic can be found in Appendix II.

## Results

Twenty-two (35%) of the 63 invited adults with CP (44% men) responded. Three did not participate; one declined, one could not be contacted and one was not able to take photos nor had access to the internet or was willing to ask others to take photos, resulting in 19 participants (32% male) who signed informed consent. They had a mean age of 57.8 years (SD 9.4) and about half of them (47%) had followed higher education. Nine participants (47%) lived with a partner and seven (37%) had children. Twelve participants (63%) had GMFCS and MACS levels I-II. The minority of the participants (37%) had unilateral CP (Table 1).

**Table 1:** Characteristics of the sample (*n* = 19) of adults with CP.

	***n*** **(%) or mean (SD; range)**
Man	6 (32%)
Age in years	57.8 (mean) [9.4 (SD); 44–79 (range)]
40–49 years	5 (26%)
50–59 years	6 (32%)
60–69 years	6 (32%)
70–79 years	2 (10%)
Educational level	
Low	4 (21%)
Medium	6 (32%)
High	9 (47%)
Marital status	
Single/widowed	10 (53%)
Married/cohabiting	9 (47%)
Having children	7 (37%)
Support at home	
No support	8 (42%)
Support	11 (58%)
Employment status	
Paid	7 (37%)
Volunteer	2 (11%)
Daycare facility	2 (11%)
None	8 (42%)
GMFCS	
I	4 (21%)
II	8 (42%)
III	4 (21%)
IV	2 (11%)
V	1 (5%)
MACS	
I	4 (21%)
II	8 (42%)
III	4 (21%)
IV	2 (11%)
V	1 (5%)
CP type	
Spastic	18 (95%)
Unilateral	6 (32%)
Bilateral	12 (63%)
Non-spastic (unilateral)	1 (5%)

*GMFCS, Gross Motor Classification System; MACS, Manual Ability Classification System; I - least severely affected, V - most severely affected*.

A total of 146 photos were received (mean (SD) 7.7 (2.6); range 4–15). Photos included mobility issues, (facilitating or hampering) physical environments, activities at the physiotherapy practice, social activities and hobbies, assistive technology devices (ATD) (e.g., walker, orthopedic shoes, sit ski) and situations or objects at home. Thematic analysis revealed four themes: (a) Meanings of citizenship; (b) Citizenship: Facilitator and barriers; (c) Paradoxes of support and participation; and (d) Future (Table 2). Analysis of interview 19 revealed no new codes with saturation in codes reached in interview 18.

**Table 2:** Summary of the themes and subthemes found in this study of adults with CP.

**Themes**	**Subthemes**
Meanings of citizenship	a. ability to participate in society (e.g., work, social activities/relationships) b. a sense of belonging (e.g., reciprocity, caring for others, family life, proud of life-achievements)
Facilitator to citizenship	a. independence and autonomy in support (e.g., agency in support and assistance, independence in mobility, physical activities)
Barriers to citizenship	a. the impact of aging (e.g., physical deterioration, needing more time to accomplish tasks) b. stigmatization (e.g., not being regarded as full) c. life-events (e.g., divorce, incapacitated for work) d. complex and time-consuming laws and regulations (e.g., difficult and multiform application processes, extended processing time)
Paradox of support and participation	a. (in)accessible contexts b. (in)sensitivity of healthcare providers (e.g., (not) listening to their needs, giving standard/personalized advices) c. (un)supportive effects of using devices (e.g., solving the pre-existing problem but sometimes provoking social exclusion and stigmatization) d. pushing on to participate and exhaustion (e.g., social roles take more effort which increases fatigue and exhaustion)
Future	a.not really thought of, yearly check up by rehabilitation specialist

### “Meanings of Citizenship”

The meaning of citizenship appeared to be ambiguous. Two participants associated citizenship with laws or social norms such as paying taxes or taking out the garbage. For others, citizenship related to their place in society. Analyses revealed two mutually influencing subthemes of what constituted citizenship for adults with CP: (a) the ability to participate and contribute to society, and (b) a sense of belonging.

*“And Agenda 22* [of the United Nations] *actually states that everyone, regardless of their disability, has the right to equal treatment. One should be able to participate in society and yes, also be fully mobile. And that's where the Netherlands lags behind quite a bit.” (Male, 53 years)*

#### Ability to Participate and Contribute to Society

Participants valued being able to participate and contribute to society in various ways (e.g., paid work and/or volunteering, sports activities and social activities); this strengthened their sense of belonging. Most participants had or have had paid employment, whether sheltered or not, or attended daycare services. This was considered to be meaningful and contributing to citizenship. Nine participants worked (or had worked) in the health, education or government sectors, three participants worked at utility companies (either banking or telecom) and one was a journalist. Participants felt responsible for their performance in work and/or volunteering and considered their efforts being meaningful for others.

Paid and unpaid work was fulfilling and attributed to citizenship. If one was not able to be involved in paid and unpaid work anymore, due to their handicap or retirement, it took time and energy to adjust to a life without work and find new meaningful daytime activities.

*“When I used to work, I was mostly focused on my work. I often did things in the evening, but not every night. And now I have a different life, I don't have to go to work anymore. Well, I found it REALLY hard to stop working.” (Female, 79 years)*

Employed participants, when energy levels sufficed, were often volunteers as well. A range of voluntary activities was seen, from ad hoc community activities to regular participation in programs or projects. Both being among other people and sharing experiences and joy, but also being able to help and support others in their lives, were the main reasons for volunteering.

*“I do the phone circle helpline on Wednesday mornings and it takes three quarters of an hour, which usually works out. And it's nice work.” (Female, 65 years)*

#### Sense of Belonging

Being part of social groups (colleagues, friends) was found to be important for participants. It contributed to their life and how they identified themselves. Longtime friendships, sometimes since high school, meant a shared biography. Participants experienced a sense of belonging by caring for others, such as their partner, children, nephews and nieces, or in community activities such as church, Lesbian Gay Bisexual Transgender (LGBT) groups, patient organizations or in a group of former rehabilitation patients.

Sense of belonging also consisted of one's biography, especially the achievements in life related to feelings of pride and self-esteem. Examples of life achievements were having a partner, (still) being able to drive a car, owning a home, sports achievements, but also having made the right choices in life. In general, participants felt that they had a vibrant personality and that they managed to have a good life.

*“Well then, if you look back at the ten years since I stopped working, those have flown by for me. In retrospect, then I've done well because that's what this whole choice was about, to have this. So if you may look back, then I think I'll give myself a pat on the back and partly thank my ex who also hammered on it very much to point of annoyance. So then I can also be grateful to him.” (Female, 49 years)*

### Citizenship: Facilitator and Barriers

**Figure 1:**
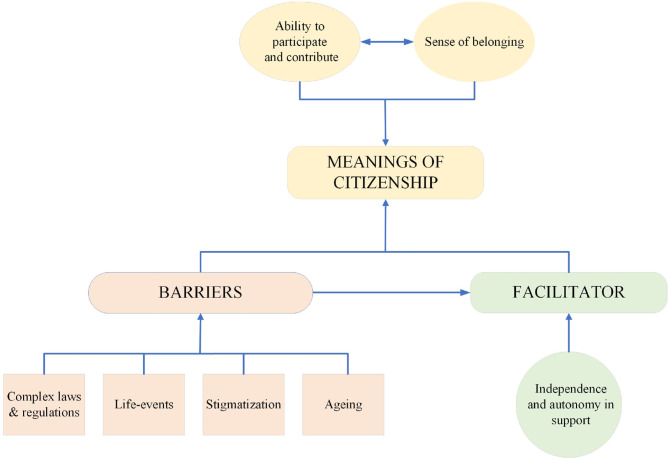
Facilitator and barriers: interrelations and relation with citizenship in adults with CP. This figure shows that “meanings of citizenship” is influenced by the “facilitator” and “barriers.” “Barriers” also affect “independence and autonomy is support.”

Citizenship could be both facilitated and hampered. Independence and agency in support formed one facilitator. The following barriers were found: (a) the negative impact of aging, (b) stigmatization, (c) life-events, and (d) complexity of laws and regulations. Some of these subthemes were interrelated (Figure 1).

#### Independence and Agency in Support

Participants indicated that when they felt heard and seen, this contributed to having agency, such as having control over how they were supported and by whom. Independence in mobility (e.g., not being dependent on adapted transport or taxi, and being allowed to go wherever one wanted to go whenever one wanted) allowed participants to socialize with others and attend events with few or no restrictions, resulting in having agency. Agency was also felt when one needed support from their partner, family or friends. However, it seemed in a lesser degree than in situations when no support was required.

*“Yes, you are different. Or you walk differently. But am I different? Do I have a problem? No. I don't see it that way. You have to make of it what you want. You have it all in your own hands. One of my hobbies is motorcycling. Well, that's all doable. But you have to do it in your own way, it might be just a little different, just like climbing stairs. You run up stairs, but I use my arms a bit more.” (Male, 48 years)*

Citizenship was something that required physical effort and it was therefore important to maintain the current level of physical functioning. For most participants, this was the motivation to exercise regularly. Examples of physical activity were cycling and walking. Exercising was performed either individually independently, organized individually, in group form in a gym, or under the guidance of a physiotherapist. Some mentioned that they were fearful of losing walking skills which would then hamper their independence.

#### Negative Impact of Aging

With aging comes physical deterioration, as manifested in problems with walking and increased spasticity. As a result, participants needed more time to accomplish their daily activities or tasks and had an increased risk of falling. As a consequence, these participants hesitated to join social events and were less confident to move around outdoors. For some participants this led to feelings of loneliness.

*“But I absolutely can't complain. At that moment it's a small moment when you see other grannies going to the playground with a grandchild. I think, well, I can't do that. Not just with him. I can't say, I'll take the bike or I'll walk to the playground and I'll let him play.” (Female, 62 years)*

For several participants increased spasticity impacted speech. One participant experienced that this complicated striking up a conversation with neighbors. If the neighbor himself was hard of hearing, social contact would be even more difficult. Speech impediments complicated being part of the local community.

#### Stigmatization

The majority of participants felt they were sometimes labeled based on their disability (e.g., spasticity, impaired speech) which then resulted in prejudices about their capabilities. Stigmatization hampered citizenship, for example when participants were not being regarded as normal. Participants were frequently approached by strangers giving them unsolicited advice. These situations felt alienating and marginalizing.

*“They think when I walk, well, oh, he must have been drinking again or something. You're being shrugged off, you've been drinking. But they never look past appearances to the inside. But I don't admit to anything either. But I don't know what to say at that moment. It's hard to explain. Yes, if I could I would, but I can't find my words either. But nobody says what I'm good at.” (Male, 64 years)*

#### Life Events

Major life events (such as being forced to stop working, death of one's spouse, divorce) were disruptive and significantly affected participants' lives (e.g., reducing their self-esteem or feeling estranged. Life events also impacted the way participants' organized their support; it forced them to apply for more support which combined with a sense of losing control over their lives. At the time of conducting the interviews, the COVID-19 pandemic was present and associated rules (e.g., keeping distance between individuals, closing communal buildings and offices, not receiving visitors) led to increased feelings of loneliness and, in some cases, even feelings of isolation.

*“A few years ago I was volunteering at* [name company] *and really needed to recover from 4 hours of volunteer work. Then I got a letter from the UWV* [Dutch benefits organization] *that stated that I had to find work because of the new Participation Act. Well, I got so angry because for 20 years I had been doing everything I was able to do, I physically can't do it anymore and now I am required to work. So I phoned them. I explained that I have volunteer work and have to do my daily naps. I'm going to talk to the occupational physician*. [UWV said then:] *You don't have to search anymore and said I would get a benefit* [instead of having to find work]. *And that was a very mixed feeling. Then I really had the feeling of failure in my life, very much so!” (Female, 44 years)*

#### Complexity of Laws and Regulations

Finally, the complex and time-consuming laws and regulations to apply for assistive technology devices (ATD), healthcare and support services were frustrating for participants. While waiting for ATD, participants felt losing agency over the situation and being at the mercy of the people who granted the approvals as well as the suppliers.

*“My work has moved to another building, how stupid can it be, with no parking spaces, ZERO. I had thought, well, two for the management, one for me and one or two for visitors, something like that, but no, ZERO! So I'm now waiting for a parking space from the city council. They've been working on that for over six months now. There is still no parking space so I go back and forth by cab. That costs me a lot of time.” (Female, 58 years)*

### Paradoxes of Support and Participation

Analyses revealed some paradoxes in the role of support and participation in the way participants experienced citizenship: (a) (in)sensitivity of healthcare providers, (b) (un)supportive effects of using devices, (c) (in)accessible contexts, and (d) pushing on to participate what leads to exhaustion. These subthemes related to citizenship directly as well as via the facilitator and barriers.

#### (In)sensitivity of Healthcare Providers

Many participants indicated that healthcare providers did not respond to their needs or gave just standard advice. Often this made participants feel unheard and unseen; they felt they were not recognized and that they were stonewalled. On the other hand, participants valued “sensitive” healthcare providers who listened to their needs and pursued an equal relationship. The physiotherapist appeared to be important in the participant's life, not only for guidance to deal with the impact of aging, but also as a life coach. The physiotherapist often had a long-term supportive relationship with participants by listening to the participant's story, empathizing with them and giving advice regarding daily challenges. About half of the participants had both positive and negative experiences with healthcare providers.

*“Well no, they didn't seem to care much about what I said. Because I remember that the orthopedist said afterwards ‘You were right, so sorry I didn't listen to that'. So they were probably using some kind of protocol or something. I hate that in healthcare. they thought they knew what I needed.”(Female, 56 years)*

*“When the shoes didn't give the right results, he said, “then we'll go into surgery.” He had come up with a plan for that. Then I went to see a neurologist. She saw the plan, we talked about it and she said “I think the orthopedist is looking too much at the foot and not at the whole person.” So she recommended that I have a gait analysis. The gait analysis showed that the intended surgery would be counterproductive. I am intensely grateful.”(Female, 56 years)*

#### (Un)supportive Effects of Using Devices

Many participants used some form of ATD in their daily lives. ATD (e.g., orthopedic shoes, walker, (electric) wheelchair) improved participants' mobility, which was supportive in engaging in community activities. ATD also positively affected the relationship with the partner, for example, an adapted electric twin bed enabled the participant to sleep with her spouse again. On the other hand, participants were sometimes reluctant to implement ATD in their daily lives to avoid possible stigmatization. For one participant, an ATD improved mobility outdoors, but she was not yet emotionally ready to be seen with this device. Others said they did not want to stand out in society using an ATD. In addition, using an ATD could result in new problems. For example, the use of a walker contributed to stability during walking, but also resulted in a crouched posture while walking which eventually caused back pain. Pain was also caused by an ankle foot orthosis: it improved walking, but hindered bicycling due to chafing of the skin when pedaling.

#### (In)accessible Contexts

The accessibility of the physical environment affected citizenship. An obstacle free physical environment allowed participants to be active in society without spending too much energy. So, accessibility not only meant *access*, it was also helping participants to maximize the degree of *engagement*.

*“…. In Spain they are everywhere in the street, well, every 50 to 100 meters there is a bench. Just a bench. So when I'm in Spain with my brother, he says, ‘oh, you want walk to the boulevard?'. Well that's about 10 to 15 minutes. That would be impossible for me to do here.” (Female, 62 years)*

Conversely, inaccessible physical environments hindered the participants in taking part in society. Participants regularly experienced poor accessibility of public buildings, shops, restaurants and public transport; for example, staircases without handrails, too small toilets or absence of train boarding assistance staff. When confronted with unexpected barriers, some felt disappointed, others felt they were not important enough or felt left out.

*“Once there was a very good film we had seen on TV, which was to be shown in the cinema. So I said ‘we are going to the cinema' and I call and I say “we use wheelchairs.” “Oh, well then it's going to be a bit different. It's not allowed.” I say “why not?' Well because of the fire hazard.” (Male, 53 years)*

#### Pushing on to Participate and Exhaustion

The pursuit of living a ‘normal' life was sometimes accompanied by hard work and intrinsically driven perseverance. In childhood, one was encouraged by parents to both try out and persevere to achieve the next level. Because of the increased impact of CP over time, participants needed more effort to keep up with their life ambitions (e.g., social activities, work) which consequently resulted in even more strain. For some participants, this resulted in burn-out, depression or quitting their job.

*“It's okay to say “hey I'm having a bad day or my body hurts”, but yeah, I wasn't raised that way. I'm always like, let's put our shoulders to the wheel, I might not always say the right thing now. No, I think I just work harder. I notice that I never cancel an appointment or when I'm so sick or in such pain, I always go, yes, always. So I think that my drive is even higher than a healthy person.”(Female, 49 years)*

### Future

The participants indicated that they would in the future like to live as independently as possible. This theme related directly to citizenship, as well as via the facilitator and barriers, as well as paradoxes of support and participation. Almost all of the participants said they did not think through what they needed in the future to constitute their citizenship. Several participants said they would probably need more help such as using ATD, and increased use of healthcare and support services. One participant, who had not seen a rehabilitation specialist since his teenage years, had recently consulted a rehabilitation specialist. This consultation provided insight into how CP affects aging (back pain due to reduced muscular strength and degeneration of spinal vertebrae). It raised awareness that pain was a signal that needed to be addressed to be able to maintain (social) functioning.

*“And so all of a sudden I came back into the picture. And for me actually, like, I never thought about it because of* [the disability being in] *my legs that my back is wearing out or something. I mean, it turned out that there was joint wear in two vertebrae, this had also been diagnosed years earlier in another hospital with in the lower part of the back*, [lumbar] *four and five I believe it was, and the intervertebral disc, that had joint wear. And in the rehab center here I had a gait analysis done.” (Male, 48 year)*

It was also mentioned that the current single family home may eventually become unsuitable, which would mean modifying the house or moving. However, being occupied with daily hassles meant that many participants had not really thought about future needs yet, and were unaware of what to expect.

*“Yes it's hard to foresee. I tend to put blinders on for a while for that and think “I'll see when it comes.” There's no point in worrying about something that might not be necessary.” (Female, 56 years)*

## Discussion

The present study aimed to gain insight into what constitutes citizenship for middle-aged and older adults with CP and how they experience their citizenship. Findings identified the ability to participate and the sense of belonging as important aspects of citizenship for these persons. Independence and agency in support contributed to citizenship, while aging, stigmatization, life events and the complexity of laws and regulations were perceived as barriers. The paradoxes, such as (in)accessible contexts and using ATD, could either facilitate or hamper citizenship.

Participants sometimes viewed citizenship as an abstract construct. Two participants referred explicitly to civil rights or democratic practices (15, 17, 18). Other aspects, such as contributing to society (16, 18) and identity, (17) were brought up by the vast majority. This partly corresponds to how persons without disability in the Netherlands conceptualize citizenship: [(31), p.221] ‘“taking responsibility” or “showing responsible behavior,” “caring for others,” and “being a member of society.” They [persons without disability] took citizenship to mean all kinds of social rather than political things.” In addition, citizenship was associated with “solidarity”, “involvement” and “responsibility” (31). The participants in our study also referred to conditions and situations in which citizenship taking place.

Our findings are in line with Yeung, Passmore and Packer (20) who reported that barrier-free contexts and belonging to society were aspects of citizenship according to young adults with CP. Interestingly, when asked to define participation adults with lifelong conditions report similar themes (32, 33). This raises the question how citizenship and participation relate, both in theory as in the lived experiences. Our results show that middle-aged and older adults with CP consider participation (“ability to participate”) as one aspect of citizenship. The International Classification of Functioning, Disability and Health (ICF) (34) defines participation as “involvement in a life situation.” Adults with lifelong disabilities consider participation to go beyond life situations: participation is also meaningful contribution, belonging, reciprocity/equality and having agency or being independent (32, 33). However, participation emphasizes the involvement in regular productive activities, (33) which may vary internationally. The adults in our study indicated that they weighed their CP in valuing citizenship, they were proud of what they had achieved in life and were aware that their CP and support services and/or use of ATD were sometimes extra barriers. Citizenship appears to be less normative than participation for adults with CP. The lived experiences of these adults also resonate the various layers of citizenship that relate to the guiding principles of the CRPD (35). Therefore, a citizenship perspective might help health and support service providers to better align with the experiences of their clients.

Taking the lived experiences as a starting point, the citizenship perspective on participation makes sense. For example, a study on the use of ATD reflected on its inclusive and exclusive consequences (36). To better understand these consequences, the concept of “passing as normal” is helpful (37). Passing as normal can be summarized as an adaptive strategy to strive for normality, and in this way to belonging. For example, one participant reflected on the use of an ankle foot orthosis (adaptive strategy) that improved walking (normality) and facilitated citizenship (inclusive consequence). Simultaneously, the use of the ankle foot orthosis hampered riding a bike and caused pain (exclusive consequences). The adults in our study gave many examples of both inclusive and exclusive consequences of support and participation. This, in combination with other barriers such as stigmatization and physical deterioration due to aging, significantly affected the ability to participate and influenced their sense of belonging.

With regard to social citizenship, autonomous citizenship and political citizenship roles, (18) middle-aged and older adults with CP provided diverse examples of how these roles were either facilitated or hampered. Some were politically active or volunteered in patient organizations while others contributed to the local community by organizing festivities or taking part in charity committees. Social and autonomous citizenship roles were frequently hindered by stigmatization, inaccessible contexts, unsupportive healthcare and support services. Contextual factors may be more important than individual characteristics in defining citizenship roles (38).

Because of the increasing impact of CP, (7, 38, 39) healthcare utilization increases with age (40). Nevertheless, health needs often remain unmet (41–43). Adults in this study had mixed experiences with healthcare and support services. Professionals who were sensitive to needs and questions were highly appreciated; these professionals took time to listen to what mattered for the participants. Sensitivity not only resulted in appropriate care delivery (supporting the ability to participate), but also acknowledged the person behind the needs (supporting the sense of belonging) (i.e., person-centered care). The adults in our study appreciated the physiotherapist as a professional to minimize physical deterioration. The physiotherapist also provided coaching in how to deal with the impact of CP on daily life. A possible explanation for this is that physiotherapy is offered in the community in the Netherlands and that many adults thus have a long-term relationship with the practitioner. In line with other studies, the adults in this study encountered healthcare providers who were not responsive to their needs. Adults with CP often experience incompetence in healthcare providers (12, 39, 44, 45) or experience stigma when they seek support (46). More generally, citizenship of adults with lifelong disabilities has been contested by changes in long-term care policies and accompanied austerities, resulting in fragmentation of healthcare and support services (18, 35, 47). Consequently, people must do more themselves and/or rely on unpaid care (48). Adults with CP are challenged to participate in their community and to find adequate healthcare and support (11, 12, 44).

### Strengths and Limitations

The present study included middle-aged and older adults with CP with a wide age range and GMFCS and MACS levels. However, females and highly educated adults were overrepresented. As a consequence, we did not fully capture the lived experiences of males and of adults with lower levels of education, which may be due to the subject matter of the study. In a representative cohort of adults with CP 42% were women, (49) however, of the 63 invited adults for this study about 56% were women. A second limitation of the study is that the explanation to the participants of the different “meanings of citizenship” may have been too directive. In a preliminary study, however, we found that the explanation was necessary because the concept of citizenship was too abstract for the participants making it difficult to take pictures of what citizenship meant to them. By summing up the many layers of citizenship in the explanation, we tried to minimize this influence. Thirdly, differences between background (sex and age) and clinical characteristics (GMFCS and laterality) regarding the found themes could not be explored because of our sample size. Lastly, we did not compare the lived experiences of middle-aged and older adults with and without CP. Therefore it remains unclear whether some findings (i.e., the impact of life events) specifically relate to living with CP or are common for the general population. Our study was not designed to compare adults with and without CP, but to gain insight into the lived experiences of middle-aged and older adults with CP. It is clear that these experiences are not only limited to the CP itself.

### Implications for Clinical Practice

Middle-aged and older adults with CP are often confronted with a lack of expertise in health providers, which stresses the need for specialized rehabilitation care for adults with CP. On the one hand there is a need for more centers of expertise, while on the other hand a health provider in the community (e.g., a physiotherapist) can help these adults to deal with the impact of CP and daily hassles. These findings highlight the importance of person-centered rehabilitation care, in which a life-course perspective (50) is adopted and that acknowledges the experiential knowledge of these adults. Person-centered rehabilitation services are encouraged to consider the three citizenship roles (18), to proactively assess contextual barriers, and to assist adults with CP in finding solutions that work for them.

Apart from clinical implications for rehabilitation, municipal services and long-term care policymakers should develop a better sensitivity to the needs of adults with disabilities (including CP). As long-term care systems vary internationally, there are differences in equity in access to support services, (51) resulting in variations in the impact on citizenship of adults with disabilities. Services should aim at better accessible contexts to facilitate the opportunities to contribute to society (social citizenship) and to tailored and person-oriented approaches with short processing times to give room for the diversity of support needs (autonomous citizenship). Political citizenship can be better supported by including persons with disabilities (including CP) in legislation and policy making and support and healthcare delivery. All these recommendations are in line with the UN Convention on the Rights of Persons with Disabilities (CRPD) (34). The reported barriers to citizenship indicate that the implementation of the CRPD requires ongoing attention.

## Data Availability Statement

The datasets presented in this article are not readily available because Transcripts are in Dutch. Although all transcripts are anonymized, Participants' stories contain personal information. Requests to access the datasets should be directed to s.r.hilberink@hr.nl.

## Ethics Statement

The studies involving human participants were reviewed and approved by Medical Research Ethics Committee Leiden The Hague Delft. The patients/participants provided their written informed consent to participate in this study.

## Author Contributions

SH and MC contributed to conception and design of the study. VH, MH, and SH wrote the Medical Research Ethical Committee study protocol. VH conducted the interviews. VH, MC, and JS performed the analysis. VH and SH wrote the first draft of the manuscript. All authors contributed to manuscript revision, read, and approved the submitted version.

## Conflict of Interest

The authors declare that the research was conducted in the absence of any commercial or financial relationships that could be construed as a potential conflict of interest.

## Publisher's Note

All claims expressed in this article are solely those of the authors and do not necessarily represent those of their affiliated organizations, or those of the publisher, the editors and the reviewers. Any product that may be evaluated in this article, or claim that may be made by its manufacturer, is not guaranteed or endorsed by the publisher.

## Appendix I

Explanations of the multiple meanings of citizenship

Citizenship is a multi-layered construct.

Citizenship means being part of society. Being part of society can be done in different ways. Participating in society can mean working, but also having and maintaining social contacts, sports, clubs, theater, visiting neighbors. Recognizing and feeling a connection with others and society is an important part of citizenship.

Another aspect of citizenship is more juridical. Every person has rights and duties. These rights and duties determine what is allowed and how we behave.

It is also important that ethical origin, religion and physical functioning can result in both differences and similarities in our norms and values. This is your own identity. Because people have different customs, have been brought up differently or do not speak the same language, every person is different. Here, citizenship means dealing with differences.

Finally, people (with and without disabilities) are encouraged to live independently and participate in society in a good way for as long as possible.

We want to study how adults with CP describe their citizenship; what it means to them. We also want to investigate what it takes to keep shaping citizenship. How does being part of society work, what does it make you or how do you feel connected to society. How do you contribute to society, how do you fulfill your civic rights and obligations? How do you deal with being different in today's diverse society. We are interested in how you experience your citizenship and how you have shaped your citizenship throughout your life. For this, we would like to have a conversation with you. The interview can take place at the rehabilitation center or at your home, whichever is most convenient for you.

## Appendix II

Infographic in English.
